# Oral Thrush: An Entity With a Diagnostic Dilemma

**DOI:** 10.7759/cureus.54916

**Published:** 2024-02-26

**Authors:** Aakanksha V Tiwari, Suwarna Dangore-Khasbage

**Affiliations:** 1 Oral Medicine and Radiology, Sharad Pawar Dental College and Hospital, Datta Meghe Institute of Higher Education and Research, Wardha, IND

**Keywords:** opportunistic infection, immunity, candidiasis, burning sensation, antibiotics

## Abstract

*Candida albicans* is a commensal found in the oral cavity. It is the most frequently encountered infection in the oral cavity which can be predisposed by a number of factors including most commonly compromised immunity, certain drugs, poor oral hygiene, and ill-fitting dentures. Clinical presentation involves whitish patches or erythema over the buccal mucosa, tongue, and palate depending on the type and occurrence of the condition. Oral physicians have a key role in the diagnosis of such lesions as they are well acquainted with their appearance and features. Prompt treatment can be advised once the lesion is diagnosed. This case report presents a male patient aged 65 years who reported to the Department of Oral Medicine and Radiology with a complaint of a burning sensation in the oral cavity for 4-5 days. On careful examination and based on the patient's past history, the diagnosis was given as acute pseudomembranous candidiasis. Candid mouth paint was advised which resolved the lesion and burning sensation completely. The primary takeaway from this case is that though candidiasis is routinely encountered in day-to-day practice, its diagnosis is usually missed due to its similarity with various other white lesions. Hence, the clinician must be acquainted well with lesions having diagnostic dilemmas as their appropriate diagnosis is crucial. Oral physicians play a vital role in cases of oral thrush in their diagnosis and accurate and prompt intervention.

## Introduction

A prevalent fungal infection of the oral cavity is oral candidiasis, and it frequently manifests in very young, geriatric, and unwell populations [1]. The *Candida albicans* species is often isolated from the oral cavity in those who are healthy as well as the diseased population. It is followed by *Candida tropicalis, Candida glabrata, Candida parapsilosis*, and *Candida krusei.* One of the dimorphic fungi that exists in both the yeast and hyphal or mycelial phases is *Candida albicans* [1]. While hyphal forms are linked to host tissue invasion, yeast is a harmless type [2]. When the host defenses are undermined, these commensals are able to enter the deeper tissues [3].

*Candida albicans* is the most common causative agent of opportunistic infection. It is a dimorphic fungus which normally exists in a state which is nonpathogenic in around 50% of healthy individuals. However, in favourable circumstances, it gets transformed into a pathogenic form that causes disease [4]. Numerous local and systemic factors have been linked to the development of candidiasis: dry mouth, poor oral hygiene, chronic trauma to the oral mucosa, use of local antimicrobials, long-term use of inhalational and topical steroids, radiation therapy to the head and neck area, iron deficiency anemia, diabetes mellitus, primary immunodeficiency, human immunodeficiency virus (HIV) infection and acquired immunodeficiency syndrome (AIDS), leukemia and other malignancies, neutropenia, use of steroids and immunosuppressive medication, broad-spectrum antibiotics, hypoparathyroidism, cortical adrenal insufficiency, and other endocrine diseases [5].

It is usual and often left misdiagnosed in the elderly population, specifically in individuals who are denture wearers. This is because of the failure of proper cleaning of denture surfaces which are in contact with the alveolar mucosa. This in turn may lead to yeast or fungal growth and may lead to infection. As the condition is painless, it is often left undiagnosed which may lead to chronicity and secondary mucocutaneous manifestations. Oral mucosal candidiasis exhibits a broad range of clinical presentations. The history and clinical symptoms are the primary bases to reach the diagnosis; further testing is only necessary in complex instances. Treatment of a candidal infection requires the identification of any local or systemic causes, and their potential resolution depends on targeted management involving the removal of etiologic factors [6].

## Case presentation

Description of the case

A 65-year-old male patient reported to the Department of Oral Medicine and Radiology at Sharad Pawar Dental College and Hospital on December 26, 2023, with a complaint of whitish patches and burning sensation in the oral cavity for 4-5 days and pain and pus discharge from the upper right back region of the jaw for 3-4 days. There was a history of extraction in the upper right back region 7-8 days back. He had a habit of smoking 2-3 packs of bidi for the past 10-12 years. He was a known case of hypertension and diabetes mellitus and for two years was on medication for the same. There was a history of pulmonary tuberculosis 8-9 years back for which the patient had taken directly observed therapy (DOT) treatment and the symptoms were completely resolved on the completion of treatment. He also gave a history of peripheral vascular disease for six months and gave a history of blackening of fingertips of the left limb for one month. He was admitted at Acharya Vinoba Bhave Rural Hospital (AVBRH), Wardha, for needful management with blackening of fingertips. Intra-arterial thrombolysis of the left upper limb was done 10 days back at AVBRH, and he was kept on antimicrobials, analgesics, antiplatelet medication, and nutritional supplements for 15 days. He was diagnosed with gangrene of fingertips for the left upper limb and was advised amputation for the same at AVBRH.

On extra-oral examination, the patient's face seemed to be grossly symmetrical and his lips were competent. Temporomandibular joint movements were bilaterally synchronous. A single lymph node was palpable on the right and left sides of size 1x1 cm, and it was firm, mobile, and non-tender on palpation.

**Figure 1:**
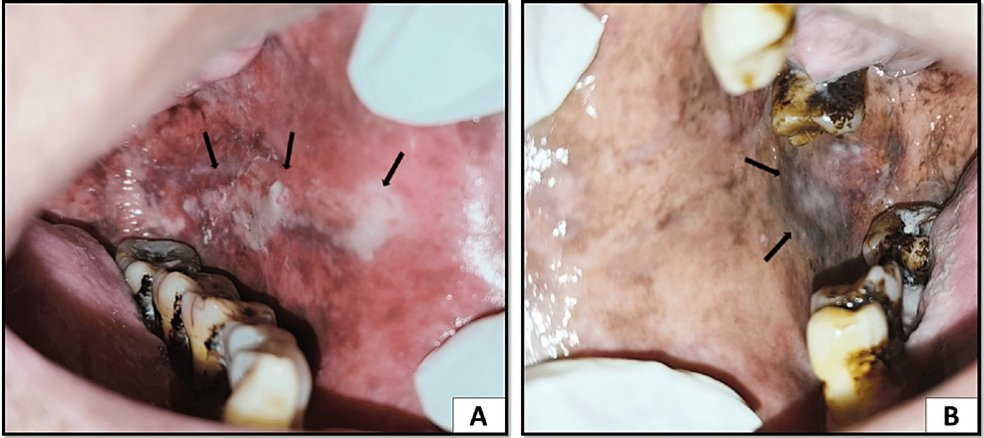
(A) Whitish raised lesion on the left buccal mucosa extending from the commissure region to the retromolar region with a curd-like appearance and generalized melanosis and erythema seen. (B) Whitish plaque present on the right buccal mucosa in the posterior region with interspersed melanosis

Careful intra-oral examination showed thick whitish plaque on the left buccal mucosa extending from the commissure region up to the retromolar region. The patch was soft, homogenous, and raised above the mucosal surface with a curd-like appearance. The lesion was scrapable using gauze and left an erythematous area behind. A similar white patch was seen on the right buccal mucosa in the posterior region which was homogenous and slightly raised above the mucosal surface. A generalized grayish-black pigmentation was present on the right and left buccal mucosa suggestive of melanosis (Figure 1).

**Figure 2:**
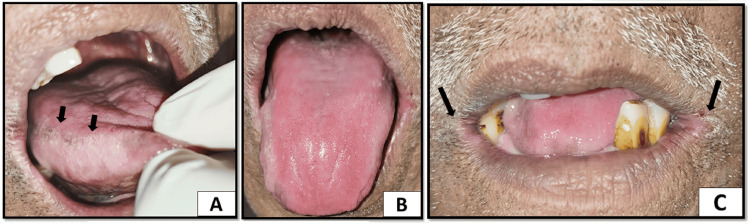
(A) Small discrete whitish patches on the right lateral border of the tongue. (B) The dorsal surface of the tongue showing an absence of filiform papillae with a smooth shiny erythematous appearance suggestive of a bald tongue. (C) Erythema and fissuring seen on the corners of the mouth suggestive of angular cheilitis

Discrete small whitish patches were present on the right lateral border of the tongue. The dorsal surface of the tongue showed a loss of filiform papillae suggestive of a bald tongue. There was evidence of fissuring and erythema on the corners of the mouth which were painful (Figure 2).

**Figure 3:**
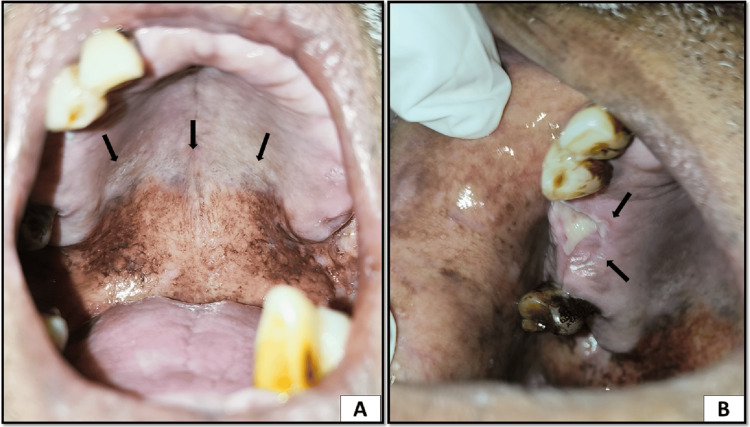
(A) Posterior region of the hard palate showing keratosis and a white cobblestone appearance suggestive of smoker's palate and severe melanosis on the soft palate. (B) Non-healing extraction socket with sloughing seen in region 16 suggestive of epulis granulomatosa

Anterior two-thirds of the hard palate had whitish patches suggestive of hyperkeratosis. In the posterior part of the palate, a white cobblestone appearance was evident with fissuring. The lesion was not scrapable. Soft palate presented severe melanosis. In the extraction socket of region 16, there was an emanating slough in the socket with surrounding localized gingival inflammation. On palpation, there was severe tenderness in region 16 (Figure 3).

Considering the clinical presentation and medical history of the patient, as the lesion on the buccal mucosa and right lateral border of the tongue was scrapable and was acute in occurrence and also there was the presence of fissuring and erythema on corners of the mouth which suggested angular cheilitis (an indication of compromised immunity), the diagnosis was made as acute pseudomembranous candidiasis. Most likely in this case, compromised immunity and antibiotic medications might be considered to be the cause of this acute opportunistic flare. As the palate revealed a white cobblestone appearance with fissuring, it was diagnosed to be a smoker's palate. Sloughing in the extraction socket suggested inflammatory response post extraction and was diagnosed to be epulis granulomatosa.

A complete stoppage of habit was advised to the patient. He was prescribed candid mouth paint for 15 days and amoxicillin plus clavulanic acid combination 625 mg twice daily along with Zerodol-SP twice daily for five days. He was already on nutritional supplements; hence, other supplements were not added. He was then recalled to the Department of Oral Medicine for review after 15 days.

**Figure 4:**
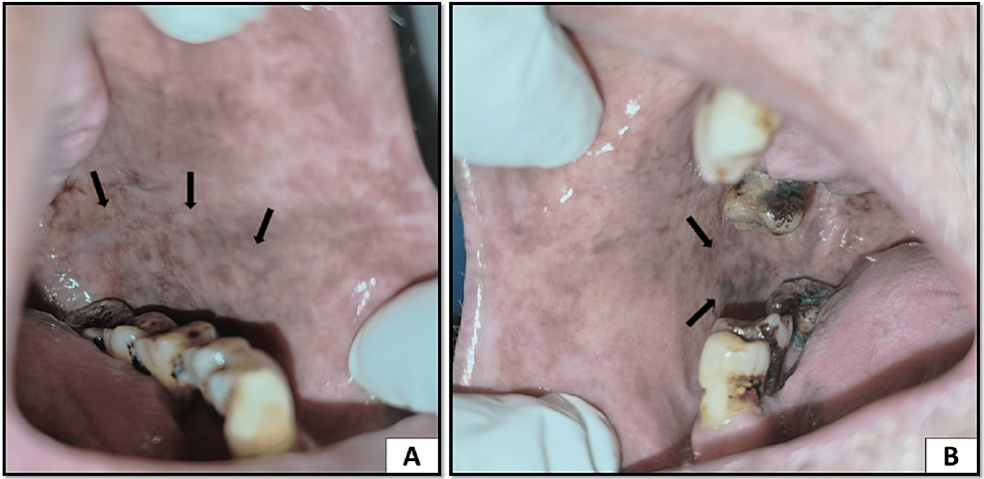
(A) Completely resolved white raised plaque on the left buccal mucosa with reduced melanosis. (B) Reduced white plaque and melanosis on the right buccal mucosa in the posterior region

On the follow-up visit, the whitish raised patch on the buccal mucosa and on the right and left buccal mucosa had been completely resolved, and there was a significant reduction in the burning sensation (Figure 4).

**Figure 5:**
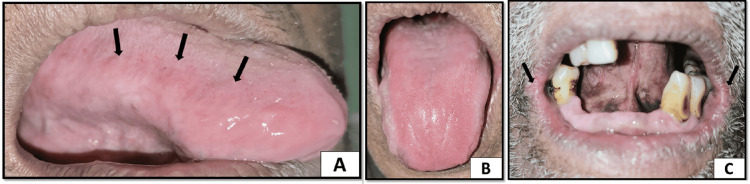
(A) Completely resolved discrete small white patches on the right lateral border of the tongue. (B) Reduced erythema on the dorsal surface of the tongue. (C) Reduced erythema and fissuring on corners of the mouth

The dorsal surface of the tongue showed reduced erythema, and the discrete white patches on the right lateral border of the tongue had been reduced. Corners of the mouth appeared to have reduced erythema and fissuring, and there was complete resolution of pain (Figure 5).

**Figure 6:**
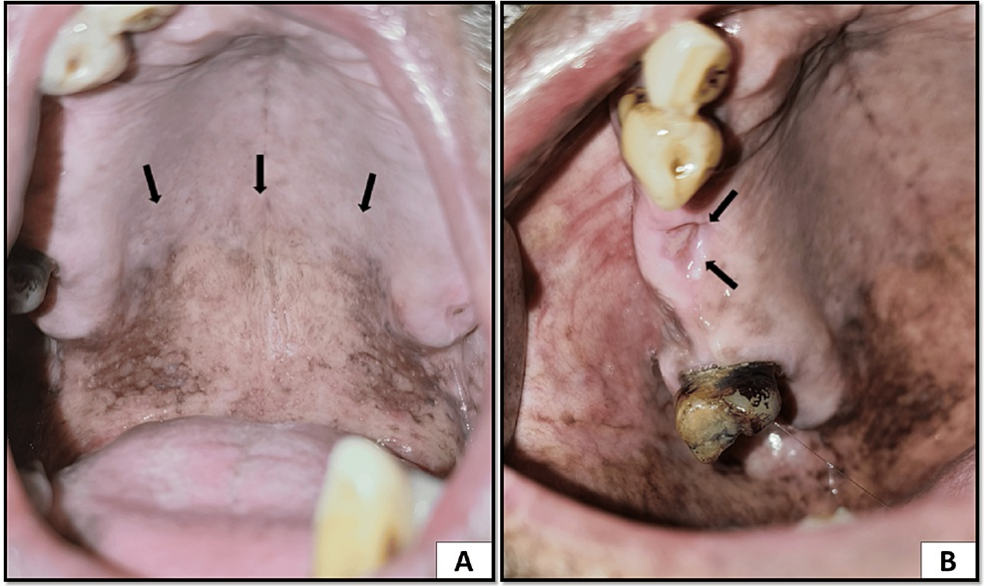
(A) Reduced keratosis and melanosis on the palate. (B) Absence of sloughing and sloping margins of the extraction socket in region 16 suggestive of healing

Palatal keratosis was also reduced along with melanosis in the posterior region. The extraction socket was also healing, and there were no slough and tenderness on palpation (Figure 6).

He was advised to continue the use of candid mouth paint for seven days and was suggested regular follow-ups every three months.

## Discussion

Candidiasis of the mouth is caused by an overabundance of the fungus *Candida* due to various underlying factors. According to reports, 20-75% of the general population carries the disease without exhibiting any symptoms [7]. The candidal adherence to the cell walls of the epithelium by a few components of fungal cell walls, including mannose, C3d receptors, mannoprotein, and saccharins, causes the infection to begin. The extent of the fungus' hydrophobicity and its capacity to attach to the host's fibronectin are also crucial during the early phases of infection. Other factors that are implicated include the creation of germ tubes, the occurrence of mycelia, perseverance in the cell walls of the epithelium, endotoxins, activation of tumor necrosis factor, and proteinases. The pathogenicity of some strains of *Candida albicans* has also been linked to their capacity for "phenotypic switching," or the ability to transition between distinct morphologic phenotypes [8].

In this case report, the patient had a systemic history and few clinical presentations like angular cheilitis which suggested a compromised immune system and a bald tongue suggesting nutritional deficiency. Also, there was a history of medications which induced flare of opportunistic infection. Smoking bidi was also thought to be one of the causative factors, but as the patient gave a history that the condition was acute, it can be considered to be an aggravating factor rather than a causative factor. Candid mouth paint worked absolutely well and the lesion was resolved and the burning sensation also subsided. It has been suggested for seven days initially so that the response to the treatment can be evaluated by reviewing the patient after one week. This is to avoid any possible side effects of the long-term use of candid mouth paint.

Patients suffering from xerostomia are more likely to experience a candidal miscarriage because their saliva does not function as a buffer and does not include antifungal substances like lysozyme and lactoferrin [6]. There is a larger concentration of yeast carriage in acidic saliva, according to a correlation between the surface pH and *Candida* carrier status [6]. According to studies, the counts are highest in the early morning [6]. The count rises as you sleep and falls when you clean your teeth and eat [6].

A theory by Arendorf and Walker has been proposed to connect smoking and candidal colonization. It is believed that smoking cigarettes causes localized epithelial changes that facilitate candidal colonization or that the smoke itself feeds *Candida albicans* [9]. However, a study by Samaranayake has found no connection between chewing betel quid and *Candida* species colonization in the mouth [10]. Candidal colonization primarily occurs on the posterior dorsum of the tongue [11]. Individuals with blood group "O" and those who do not secrete antigens of blood groups in their saliva exhibit higher levels of species of *Candida* [11]. Certain antibodies against *Candida albicans* and declining T-lymphocyte helper-to-suppressor ratios also have an impact on carriage rate [11].

As seen in Table 1, oral candidiasis is primarily divided into primary and secondary infections [12].

**Table 1:** Classification of candidiasis as primary and secondary AIDS: acquired immunodeficiency syndrome

Primary oral candidiasis	Secondary oral candidiasis
Acute form: pseudomembranous; erythematous	Oral manifestation of systemic mucocutaneous candidiasis: familial mucocutaneous candidiasis; diffuse chronic mucocutaneous candidiasis; familial mucocutaneous candidiasis; chronic granulomatous disease; candidiasis endocrinopathy syndrome; AIDS
Chronic form: erythematous; pseudomembranous; nodular; plaque-like	
*Candida-*associated lesions: denture stomatitis; angular cheilitis; median rhomboid glossitis	

The differential diagnosis of candidiasis most frequently includes oral mucosal lesions that manifest as white lesions such as leukoedema, chemical burns, lichen planus, candidiasis, leukoplakia, frictional keratosis, and white sponge nevus [13]. A small percentage of these lesions, such as burns caused due to certain chemicals and candidiasis, can be scraped, whereas the majority are frequently not [13]. The mouth's angles are impacted by angular stomatitis, also known as angular cheilitis or perleche, which can cause erythema, fissuring, and discomfort [13]. Bacteria and yeast are examples of predisposing factors. It is believed that in people with lower face height from old age or edentulous arch, the lesion arises from maceration at the mouth angles from deep, occlusive folds of the skin [13].

There is debate over the function that *Candida* plays in the causation of malignancy of the oral cavity [1,2]. The development of nitrosamine compounds by certain *Candida *species may be linked to carcinogenesis [1,2]. These chemicals can have a direct action over the mucosa of the oral cavity or may have an interaction with some other chemical carcinogens in order to trigger specific protooncogenes, hence causing oral neoplasia [1,2]. Cigarette smoke contains hydrocarbons that can activate the cascade of enzymes of *Candida*, increasing hydrocarbon's carcinogenic activity [1,2]. As a result, candidal leukoplakia may be more susceptible to malignant transformations than other types of leukoplakia [1,2].

The first stage in making a diagnosis is a detailed examination of the oral cavity and relevant history. Clinical signs and symptoms, a positive cytological smear, staining with 10% potassium hydroxide, culture on Sabouraud's dextrose agar, germ tube test, and tissue biopsy are the key factors used to make the diagnosis. More than 400 CFU/ml of saliva indicates a higher likelihood of oral candidiasis [14]. Additionally, in cases with hyperplastic candidiasis, a biopsy may be indicated. Additionally, CHROMagar Candida® culture can be used to identify specific *Candida* species [14]. Immunological and genetic methods, such as polymerase chain reaction and enzyme-linked immunosorbent test, can also be utilized to isolate and diagnose various species of *Candida* [14].

Oral candidiasis can be topically treated with a variety of antifungal medications. Topical treatments include clotrimazole troches applied five times a day for 14 days and nystatin solution [15]. A minimum of two minutes of contact time in a proper way between the oral mucosa and topically applicable agents is necessary [15]. Treatment needs to be continued for two to three days after the last clinical indications and symptoms [15]. Various medications, including clotrimazole, could be given orally as a suspension and amphotericin B as lozenges [15]. Patients who are severely immunocompromised and locally dispersed and whose response to the first line of treatment is inadequate may benefit from second-line medications such as itraconazole, ketoconazole, and fluconazole [15]. Regular follow-up and topical antifungal mouthwash were recommended for the current instance [15].

Debbarma et al. in their case report presented a male patient aged 56 years who came with a chief complaint of ill-fitting denture. He was diagnosed incidentally with chronic hyperplastic and erythematous candidiasis. The patient was given two daily doses of chlorhexidine mouthwash, two daily doses of miconazole oral gel, two daily doses of 200 mg ketoconazole tablets, two daily doses of 100 mg vitamin C, and two daily doses of 100 mg vitamin B complex for 14 days [16].

Ohashi et al. reported an old man aged 75 years, with pneumonia associated with coronavirus infection, who was admitted to the hospital. Following hospitalization, lopinavir/ritonavir was given orally, and ciclesonide was breathed for seven days. A whitish-raised patch was discovered in his oral cavity on the 14th day of his hospital stay. Oral bacterial tests were used to identify *Candida albicans*, and amphotericin B was then started. The *Candida albicans-*negative result was confirmed on the almost 35th hospital day. Dental professionals' intraoral monitoring and intervention are thought to be crucial in preventing infectious problems brought on by corticosteroids [17].

The primary takeaway from this case is that though candidiasis is routinely encountered in day-to-day practice, its diagnosis is usually missed due to its similarity with various other white lesions. Hence, the clinician must be acquainted well with lesions having diagnostic dilemmas as their appropriate diagnosis is crucial. Oral physicians play a vital role in cases of oral thrush in their diagnosis and accurate and prompt intervention. Many entities can be diagnosed clinically alone as they have typical clinical presentation as seen in the case discussed above; certain lesions may require histopathological evaluation to rule out dysplasia. Management targeted specifically to diagnose lesions leads to the complete resolution of symptoms in a few weeks. Finally, educating the patient about the cessation of adverse habits, if any, is important [18].

## Conclusions

Oral thrush is accurately said to be an infection of opportunity, as it occurs in the oral cavity as a commensal but causes flare due to various factors, the most common being compromised immunity, certain drugs, ill-fitting dentures, and so on. As it also presents as a whitish lesion in the oral cavity on the buccal mucosa, tongue, and palate, it is necessary to inspect any white lesions with suspicion. Although candidiasis is a very frequent oral lesion, it is typically not considered a first option, particularly if there is a positive tobacco history. Therefore, in such a situation, a thorough history and careful clinical evaluation tests are essential. Clinicians must be well acquainted with all forms of candidiasis and their presentations so as to conclude an accurate diagnosis. Oral physicians have a significant role to diagnose and treat such lesions. In the presented case, detailed history and clinical examination helped to arrive at a definite diagnosis without any histopathological evaluation, and prompt and targeted management led to the resolution of almost most of the symptoms in only seven days.
